# Relating Entropies of Quantum Channels

**DOI:** 10.3390/e23081028

**Published:** 2021-08-10

**Authors:** Dariusz Kurzyk, Łukasz Pawela, Zbigniew Puchała

**Affiliations:** 1 Institute of Theoretical and Applied Informatics, Polish Academy of Sciences, ul. Bałtycka 5, 44-100 Gliwice, Poland; dkurzyk@iitis.pl (D.K.); z.puchala@iitis.pl (Z.P.); 2 Faculty of Physics, Astronomy and Applied Computer Science, Jagiellonian University, ul. Łojasiewicza 11, 30-348 Kraków, Poland

**Keywords:** quantum channels, random matrices, entropies of quantum states

## Abstract

In this work, we study two different approaches to defining the entropy of a quantum channel. One of these is based on the von Neumann entropy of the corresponding Choi–Jamiołkowski state. The second one is based on the relative entropy of the output of the extended channel relative to the output of the extended completely depolarizing channel. This entropy then needs to be optimized over all possible input states. Our results first show that the former entropy provides an upper bound on the latter. Next, we show that for unital qubit channels, this bound is saturated. Finally, we conjecture and provide numerical intuitions that the bound can also be saturated for random channels as their dimension tends to infinity.

## 1. Introduction

One of the important areas of quantum information theory refers to an entropic picture of quantum states and operations. It is well known that the entropic uncertainty principle can be applied in quantum key distribution protocols [1,2] in order to quantify the performance of these protocols. Another possible area where such an approach prevails is resource theory [3]. The entropic approach in the description of quantum states can also be useful in studies of quantum phenomena such as correlations or non-locality [4,5,6,7]. Another essential aspect of quantum information theory is studying the time evolution of quantum systems interacting with the environment. Entropic characterization of quantum operations can be helpful in the investigation of decoherence induced by quantum channel [8,9] and in the study of quantum testers [10]. There exists also numerous approaches to the formulation of entropic uncertainty principles [11,12,13,14,15], which can be useful in the analysis of quantum key distribution, quantum communication or characterization of generalized measurements. In [8,9] entropy of quantum channel is defined as the entropy of the state corresponding to the channel by the Jamiołkowski isomorphism.

In quantum information theory, the relative entropy D(ρ∥σ)=Tr(ρlogρ−ρlogσ) plays an important role [16] and can be useful in quantifying the difference between two quantum states. In terms of quantum distinguishability, relative entropy can be interpreted as a distance between two quantum states. Nevertheless, it is crucial to remember that it is not a metric as it does not fulfill the triangle inequality. It can be noticed that quantum transformation Φ cannot increase of a distinguishability between quantum states ρ, σ what can be written as $D\left(\rho ∥\sigma \right)\geq D\left(\Phi (\rho )∥\Phi (\sigma )\right)$. This fact, sometimes called the data processing inequality plays an important role in the context of hypothesis testing [17]. The quantum relative entropy can also be useful in the quantification of quantum entanglement. In this context, the amount of the entanglement of a quantum state ρ can be defined as an optimal distinguishability of the state ρ from separable states e.g., $\min_{\sigma \in \mathrm{SEP}}D\left(\rho ||\sigma \right)$, where minimization is performed over separable states. It can be noticed that the relative entropy can also be used to define of von Neumann entropy of ρ as S(ρ)=logd−D(ρ∥1l/d). This definition shows that the entropy of a quantum state is related to its distance from the maximally mixed state.

The first approach to defining the entropy of a quantum channel was proposed by Życzkowski [8], where the entropy of a quantum operation was defined as the von Neumann entropy of its corresponding Choi–Jamiołkowski state. A decade later, the approach discussed in the previous paragraphs, useful for defining the entropy of quantum states was utilized by Gour and Wilde [18], where relative entropy of quantum channels was introduced. According to their approach, the von Neumann entropy of the quantum channel is given by an optimized relative entropy of the output from an extended channel relative to the output of the extended depolarizing channel. The optimization is performed over all possible input states. Moreover, there is also a possibility to define other information measures, e.g., conditional entropy or manual information, in terms of relative entropy. Recently the relative entropy of quantum channel and its generalizations are used in resource theory [19], studies of quantum channels e.g., distinguishability [20], quantum channel discrimination [21] or channel capacity [22,23].

## 2. Preliminaries

Let X, Y denote complex Euclidean spaces, let dim(X) denote the dimension of the space X and L(X,Y) denotes a set of linear operators from X to Y. For simplicity we will write L(X)≡L(X,X). If ρ∈L(X) is Hermitian ($\rho =\rho^{†}$), positive semi-definite (ρ≥0) and a trace-one (Trρ=1) linear operator then ρ is called as density operator. To keep our expressions simple, we will write the density operator corresponding to a pure states as lowercase Greek letters ϕ≡|ϕ〉〈ϕ|. In order to keep track of subspaces of composite systems we will write $\rho_{AB}\in L\left(X_{A}⊗X_{B}\right)$.

The set of all such mapping Φ:L(X)→L(Y) will be denoted by T(X,Y) and for brevity, we will write T(X)≡T(X,X). A mapping that is completely positive and trace-preserving is called a quantum channel. The set of all quantum channels will be denoted C(X,Y). There exists a well-known bijection between the sets C(X,Y) and L(Y⊗X), the Choi–Jamiołkowski isomorphism. It is given by the relation

(1) $$D_{\Phi }=\left(\Phi ⊗1\;l\right)\left(\phi^{+}\right)$$

where $|\phi^{+}\rangle =\sum_{i}^{\dim X}|ii\rangle$ and $D_{\Phi }$ is called the dynamical matrix or Choi matrix. Normalized $D_{\Phi }$ known as the Choi–Jamiołkowski state. It will be denoted as $J_{\Phi }=D_{\Phi }/\mathrm{Tr}D_{\Phi }$.

The von Neumann entropy of ρ∈L(X) is defined by the following formula

(2) H(ρ)=−Trρlogρ,

similarly to the classical Shannon entropy. This equation can be rewritten using the notion of relative entropy, which is defined for states ρ and σ analogously to its classical counterpart [24].

(3) D(ρ∥σ)=Trρ(logρ−logσ).

Here we use the convention that D(ρ∥σ) is finite when supp(ρ)⊆supp(σ). Otherwise, we put D(ρ|σ)=∞. Thus, we can rewrite Equation (2) as

(4) $$H\left(\rho \right)=\log\dim\left(X\right)-D\left(\rho ∥\rho_{*}\right),$$

where $\rho_{*}=1\;l/\dim\left(X\right)$.

The definition of quantum relative entropy can be extended to the case of quantum channels in the following manner [18].

(5) $$D\left(\Phi ∥\Psi \right)=\underset{\rho_{AR}\in L\left(X_{A}⊗X_{R}\right)}{\sup}D\left(\left(\Phi ⊗1\;l\right)\left(\rho_{AR}\right)∥\left(\Psi ⊗1\;l\right)\left(\rho_{AR}\right)\right).$$

The state $\rho_{AR}$ can be chosen as a pure state and the space $X_{R}$ can be isomorphic to $X_{A}$. Utilizing Equation (5) we get the following definition of the entropy of a quantum channel

**Definition** **1** ([18]). *Let $\Phi \in C(X_{A},X_{B})$. Then its entropy H(Φ)is defined as*

(6) $$H\left(\Phi \right)=\log\dim X_{B}-D\left(\Phi ||R\right),$$

*where $R\in C(X_{A},X_{B})$ is the depolarizing channel $R:\rho \mapsto \left(\mathrm{Tr}\rho \right)\;1\;l/\dim\left(X_{B}\right)$.*

The quantum entropy was also defined in the same matter in [17]. However, there exists an earlier definition of entropy of a quantum channel. In [8,9], the quantum channels was characterized by the map entropy, which was defined as the entropy of corresponding Jamiołkowski state. It reads

**Definition** **2** ([8]). *Let $\Phi \in C(X_{A},X_{B})$. Its entropy H(Φ) is given by the entropy of the corresponding Choi-Jamiołkowski state*

(7) $$H^{K}\left(\Phi \right)=H\left(J_{\Phi }\right).$$

The above entropy achieves its minimal value of zero for any unitary channel and the maximal value of $2\log\dim X_{B}$ for the completely depolarizing channel. Based on these two definition we arrive at the following observation.

**Lemma** **1.** *Let $\Phi \in C(X_{A},X_{B})$. The two possible definitions of quantum channel entropy H(Φ) and $H^{K}\left(\Phi \right)$ fulfill the following relation*

(8) $$H\left(\Phi \right)\leq H^{K}\left(\Phi \right)-\log\dim X_{B}$$

**Proof.**  The proof follows from a direct inspection

(9) $$H\left(\Phi \right)=\log\dim X_{B}-\underset{|\psi \rangle \in X_{A}⊗X_{R}}{\sup}D\left((\Phi ⊗1\;l)(\psi )∥(R⊗1\;l)(\psi )\right).$$

Let us denote $\sigma_{BR}=\left(\Phi ⊗1\;l\right)\left(\psi \right)$. Now we note that $\left(R⊗1\;l\right)\left(\psi \right)=1\;l/\dim\left(X_{B}\right)⊗{\mathrm{Tr}}_{A}\psi$ and we use the well known identity log(1l⊗ρ)=1l⊗logρ and we have

(10) $$H\left(\Phi \right)=\log X_{B}-\underset{|\phi \rangle \in X_{A}⊗X_{R}}{\sup}\left(-H\left(\sigma_{BR}\right)-\mathrm{Tr}\sigma_{BR}\left(\frac{1\;l}{\dim(X_{B})}⊗\log{\mathrm{Tr}}_{A}\psi \right)\right).\;$$

Finally we note that $\mathrm{Tr}\sigma_{BR}\left(1\;l⊗\log({\mathrm{Tr}}_{A}\psi )\right)=\mathrm{Tr}{\mathrm{Tr}}_{B}\sigma_{BR}\log{\mathrm{Tr}}_{A}\psi$ and ${\mathrm{Tr}}_{B}\sigma_{BR}={\mathrm{Tr}}_{A}\psi$. Putting this into Equation (10) along with the fact that $J_{\Phi }$ is normalized we get the desired result. □

The main focus of this work is to find instances that saturate the inequality in Lemma 1. We will mainly focus on the study of unital qubit channels.

## 3. Quantum Unital Qubit Channels

In this section we will focus our attention on unital qubit channels, that is $\Phi \in C(C^{2})$ such that Φ(1l)=1l. Our goal here is to show that the supremum present in Equation (5) is achieved for the maximally entangled state $|\phi^{+}\rangle$. This can be formally written as the following theorem

**Theorem** **1.** *Let $\Phi \in C(C^{2})$, such that Φ(1l)=1l. Then*

(11) $$H\left(\Phi \right)=H^{K}\left(\Phi \right)-\log2.$$

We need to stress here that a non-unital quantum channel Φ still might achieve $H\left(\Phi \right)=H^{K}\left(\Phi \right)-\log\dim X_{B}$. Let us consider as a simple example a channel which creates an arbitrary state σ

(12) $$\Phi_{\sigma }\left(X\right)=\mathrm{Tr}\left(X\right)\sigma .$$

We observe that for any input state $\rho_{AB}\in Ł\left(X_{A}⊗X_{B}\right)$ it holds that

(13) $$\begin{array}{cc} \; & D\left(\left(\Phi_{\sigma }⊗1\;l\right)\left(\rho_{AB}\right)∥\left(R⊗1\;l\right)\left(\rho_{AB}\right)\right)=\\ = & D\left(\sigma ⊗\rho_{B}∥1\;l/\dim\left(X_{B}\right)⊗\rho_{B}\right)=D\left(\sigma ∥1\;l/\dim\left(X_{B}\right)\right). \end{array}$$

Hence, the supremum in Equation (5) is achieved for any input state. In particular, we might choose it to be the maximally entangled state $\phi^{+}$, which gives us the equality. In fact, the equality will hold when the supremum is achieved for a maximally entangled state.

The remainder of this section contains technical lemmas which combined give the proof of Theorem 1.

A generic two-qubit state can be written as

(14) $$|\psi_{AR}\rangle =U⊗V(\sqrt{p}|00\rangle +\sqrt{1-p}|11\rangle ),$$

for some qubit unitary matrices U,V. Let us note that the quantum relative entropy is unitarily invariant $D\left(\rho ∥\sigma \right)=D\left(U\rho U^{†}∥U\sigma U^{†}\right)$. Moreover, we use the same the fact that the Jamiołkowski matrix of channel $\Phi_{W}\left(\rho \right)=\Phi \left(W\rho W^{†}\right)$ has the same spectrum as the Jamiołkowski matrix of channel Φ, where *W* is a unitary matrix. Thus, we can skip the unitary operations in our further investigations. We may perform the optimization taking into account only the parameter *p* which quantifies the amount of entanglement between the input qubits. In order to further simplify notation we will write

(15) $$|\psi_{AR}\rangle =\sqrt{p}|00\rangle +\sqrt{1-p}\left|11\rangle =\right|\sqrt{P}\rangle \rangle$$

where |X〉〉 denotes the vectorization of the matrix *X* and $\sqrt{P}=\mathrm{diag}\left(\sqrt{p},\sqrt{1-p}\right)$ and we define

(16) $$\phi_{P}=|\sqrt{P}\rangle \rangle \langle \langle \sqrt{P}|$$

In the next step we will check the symmetry of D(Φ∥R) with respect to the parameter *p*. Hence, we formulate the first lemma.

**Lemma** **2.** *Let $\Phi \in C(C^{2})$ and let $\phi_{P}$ be a two-qubit state as in Equation (16). Then D(Φ∥R) is symmetric in the parameter p.*

**Proof.**  Let us denote Q=diag(1−p,p). It can be checked that

(17) $${\left(\left(\sigma_{y}⊗\sigma_{y}\right)\left(1\;l⊗\sqrt{P}\right)D_{\Phi }\left(1\;l⊗\sqrt{P}\right)\left(\sigma_{y}⊗\sigma_{y}\right)\right)}^{*}=\left(1\;l⊗\sqrt{Q}\right)D_{\Phi }\left(1\;l⊗\sqrt{Q}\right),$$

where $\sigma_{y}$ is the Pauli matrix

$$\sigma_{y}=\left(\begin{array}{cc} 0 & -i\\ i & 0 \end{array}\right).$$

This observation combined with the fact

(18) $$\left(\Phi ⊗1\;l\right)\left(\phi_{P}\right)=\left(1\;l⊗\sqrt{P}\right)D_{\Phi }\left(1\;l⊗\sqrt{P}\right),$$

gives the symmetry of the entropy

(19) $$H\left(\left(\Phi ⊗1\;l\right)\left(\phi_{P}\right)\right)=H\left(\left(\Phi ⊗1\;l\right)\left(\phi_{Q}\right)\right).$$

As for the term $\mathrm{Tr}\left(\left(\Phi ⊗1\;l\right)\left(\phi_{P}\right)\log\left(R⊗1\;l\right)\left(\phi_{P}\right)\right)$ observe that

(20) $$\log\left(\left(R⊗1\;l\right)\left(\phi_{P}\right)\right)=1\;l⊗\log\left(P/2\right).$$

Finally

(21) $$\left(\sigma_{y}⊗\sigma_{y}\right)\left(\log\left(\frac{1}{2}1\;l⊗P\right)\right)\left(\sigma_{y}⊗\sigma_{y}\right)=\log\left(\frac{1}{2}1\;l⊗Q\right).$$

Combining all of these observations yields the lemma. □

Subsequently, we prove in next lemma the concavity D(Φ∥R) with respect to the parameter *p*.

**Lemma** **3.** *Given a unital channel $\Phi \in C(C^{2})$ and let $\phi_{P}$ be a two-qubit state as in Equation (16). Then the function $f\left(p\right)=D\left(\left(\Phi ⊗1\;l\right)\left(\phi_{P}\right)\left|\right|\left(R⊗1\;l\right)\left(\phi_{P}\right)\right)$ is concave.*

**Proof.**  For the purpose of this proof let us denote $\rho \left(p\right)=\left(\Phi ⊗1\;l\right)\left(\phi_{P}\right)$. Let us also denote

(22) $$\begin{array}{cc} g(p)= & \mathrm{Tr}\rho (p)\log\rho (p),\\ l(p)= & \mathrm{Tr}\rho \left(p\right)\log\left(R⊗1\;l\right)\left(\phi_{P}\right). \end{array}$$

A direct calculation shows that l(p)=h(p)+log2, where h(p)=−plogp−(1−p)log(1−p) is the point entropy. From this it follows that

(23) $$\frac{d^{2}l}{dp^{2}}=-\frac{1}{p(1-p)}<0.$$

For g(p) we calculate

(24) $$\begin{array}{cc} \frac{dg}{dp}= & \mathrm{Tr}\left(\rho^{\prime }\left(p\right)\log\rho \left(p\right)\right)+\mathrm{Tr}\left(\rho \left(p\right)\frac{d}{dp}\left(\log\rho (p)\right)\right)\\ = & \mathrm{Tr}\left(\rho^{\prime }\left(p\right)\log\rho \left(p\right)\right)+\mathrm{Tr}\left(\rho \left(p\right)\rho^{-1}\left(p\right)\rho^{\prime }\left(p\right)\right). \end{array}$$

Observing that $\rho^{\prime }\left(p\right)=\sqrt{J_{\Phi }}\left(1\;l⊗\rho \left(p\right)\right)\sqrt{J_{\Phi }}$ we see that the second term in Equation (24) is equal to zero. Hence, we have

(25) $$\frac{d^{2}g}{dp^{2}}=\mathrm{Tr}\left(\rho^{\prime }\left(p\right)\frac{d}{dp}\left(\log\rho \left(p\right)\right)\right).$$

From Taylor expansion of derivative formulae for matrix logarithms [25] we get

(26) $$\frac{d}{dt}\log\rho \left(p\right)=\int_{0}^{1}{\left(s(\rho (p)-1\;l)+1\;l\right)}^{-1}\rho^{\prime }\left(p\right){\left(s(\rho (t)-1\;l)+1\;l\right)}^{-1}ds.$$

Thus,

(27) $$\begin{array}{cc} \frac{d^{2}g}{dp^{2}}= & \mathrm{Tr}\left(\rho^{\prime }\left(p\right)\int_{0}^{1}{\left(s\left(\rho (p)-1\;l\right)+1\;l\right)}^{-1}\rho^{\prime }\left(p\right){\left(s\left(\rho (p)-1\;l\right)+1\;l\right)}^{-1}ds\right)\\ \leq & \mathrm{Tr}\left(\rho^{\prime }{\left(p\right)}^{2}\int_{0}^{1}{\left(s\left(\rho (p)-1\;l\right)+1\;l\right)}^{-2}ds\right). \end{array}$$

Now we will focus on the last integral above,

(28) $$\begin{array}{cc} \int_{0}^{1}{\left(s\;\rho (p)+(1-s)\;1\;l\right)}^{-2}ds & =U\left(\int_{0}^{1}{\left(s\;\lambda (A)+(1-s)\;1\;l\right)}^{-2}ds\right)U^{†}=\\ \; & =U\lambda^{-1}\left(\rho \right)U^{†}=\rho {\left(p\right)}^{-1}, \end{array}$$

where λ(ρ) is a diagonal matrix with eigenvalues of ρ on a diagonal and *U* is a unitary matrix. According to the above considerations

(29) $$\begin{array}{cc} \frac{d^{2}g}{dp^{2}}\leq & \mathrm{Tr}\left({\left(\rho^{\prime }\left(p\right)\right)}^{2}\rho^{-1}\left(p\right)\right)\\ = & \mathrm{Tr}\sqrt{D_{\Phi }}\left(1\;l⊗\rho (p)\right)\sqrt{D_{\Phi }}{\left(\sqrt{D_{\Phi }}\right)}^{-1}{\left(1\;l⊗P\right)}^{-1}{\left(\sqrt{D_{\Phi }}\right)}^{-1}\sqrt{D_{\Phi }}\left(1\;l⊗\rho (p)\right)\sqrt{D_{\Phi }}\\ = & \mathrm{Tr}J\left(1\;l⊗\rho \left(p\right)P^{-1}\rho \left(p\right)\right)=\mathrm{Tr}\left({\mathrm{Tr}}_{A}J\right)\rho \left(p\right)P^{-1}\rho \left(p\right)=\mathrm{Tr}P^{-1}=\frac{1}{p(1-p)}. \end{array}$$

Combining this with Equation (23) we see that f(p) is concave. □

Based on the above lemmas, it can be concluded that supremum in D(Φ∥R), where Φ(1l)=1l, is obtained for $p=\frac{1}{2}$, which indicates it is achieved for the maximally entangled state $|\phi^{+}\rangle$. Thus, combination of the lemmas proves Theorem 1.

## 4. Asymptotic Case

In this section, we will show that Equation (8) is saturated in the case of large system size. Firstly, let us denote $\dim\left(X_{A}\right)=\dim\left(X_{B}\right)=d$. Numerical investigations lead us to formulate the following conjecture.

**Conjecture** **1**. Let Φ∈C(X) and d=dim(X). Then as d→∞

(30) $$H\left(\Phi \right)≃H^{K}\left(\Phi \right)-\log d≃\log d-\frac{1}{2}+o\left(1\right),$$

*where* Φ *chosen randomly according to measures introduced in [26].*

To provide some intuition behind this conjecture, we first state a theorem which tells us about the distribution of eigenvalues of the output of an extended random quantum channel, when the input is also chosen randomly.

In order to properly state the theorem, we will utilize the notion of free multiplicative convolution of two distributions, μ and ν, denoted μ⊠ν. This convolution is defined for two independent random Hermitian matrices *A* and *B*, such that at least one of them is invariant, under conjugation by unitaries. If limiting eigenvalue distributions of the aforementioned matrices are μ and ν respectively, then free multiplicative convolution gives us the asymptotic distribution of eigenvalues of the product AB. For a formal definition and an algorithm for calculating μ⊠ν we refer the reader to [27].

**Theorem** **2.** *Let Φ be a random channel with Jamiołkowski matrix $D_{\Phi }$, we assume that the limiting distribution of eigenvalues of $D_{\Phi }$ is given by μ. Let ϕ be a random pure state with the limiting distribution of Schmidt values given by ν. We define*

(31) σ=(Φ⊗1l)(|ϕ〉〈ϕ|),

*then the limiting distribution of eigenvalues of σ is given by μ⊠ν.*

**Proof.**  Note that

(32) $$|\phi \rangle =\left(W⊗1\;l\right)\sum_{i}\sqrt{\lambda_{i}}|i,i\rangle ,$$

where *W* is a random unitary matrix and {|i〉} is the computational basis.

(33) $$\begin{array}{cc} \sigma =(\Phi ⊗1\;l)(|\phi \rangle \langle \phi |)\; & =\sum_{ij}\sqrt{\lambda_{i}\lambda_{j}}\left(\Phi ⊗1\;l\right)(\left(W⊗1\;l\right)|i,i\rangle \langle j,j|\left(W^{†}⊗1\;l\right))\\ \; & =\sum_{ij}\sqrt{\lambda_{i}\lambda_{j}}\left(\Phi_{W}⊗1\;l\right)(|i,i\rangle \langle j,j|), \end{array}$$

where $\Phi_{W}\left(\rho \right)=\Phi \left(W\rho W^{†}\right)$, note that Jamiołkowski matrix of channel $\Phi_{W}$ has the same spectrum that the Jamiołkowski matrix of channel Φ. Next we write

(34) $$\begin{array}{cc} \sigma & =\sum_{ij}\sqrt{\lambda_{i}\lambda_{j}}\left(\Phi_{W}⊗1\;l\right)(|i,i\rangle \langle j,j|)\\ \; & =\sum_{ij}\sqrt{\lambda_{i}\lambda_{j}}\Phi_{W}(|i\rangle \langle j|)⊗\left|i\rangle \langle j\right|\\ \; & =\left(1\;l⊗\mathrm{diag}\left(\sqrt{\lambda }\right)\right)D_{\Phi_{W}}\left(1\;l⊗\mathrm{diag}\left(\sqrt{\lambda }\right)\right). \end{array}$$

Now note, that the eigenvalues of σ are the same as eigenvalues of $D_{\Phi_{W}}\left(1\;l⊗\mathrm{diag}\left(\lambda \right)\right)$, which gives the result. □

Now, we have the following intuition behind our conjecture. Combining the results from [28,29] with [26,30] we have for large *d* and uniform distribution of channels

(35) $$H^{K}\left(\Phi \right)=2\log d-\frac{1}{2}+o\left(1\right).$$

Next, we have the following result. Let |ϕ〉 be a random pure state with the Schmidt numbers chosen according to some measure ν and let |ϕ〉 be free from Φ. Then the output state has its spectrum given by the free multiplicative convolution μ⊠ν, where μ is the distribution of eigenvalues of $D_{\Phi }$.

Let us consider following optimization target

(36) $$D\left(\Phi ∥R\right)=\underset{|\phi \rangle \in L\left(X_{A}⊗X_{R}\right)}{\sup}D\left((\Phi ⊗1\;l)(|\phi \rangle \langle \phi |)∥(R⊗1\;l)(|\phi \rangle \langle \phi |)\right),$$

where $|\phi \rangle =\left(U⊗V\right)\sum_{i}\sqrt{\lambda_{i}}|i,i\rangle$ for some unitary matrices *U*, *V*. Note that optimization result is invariant under local operations *U*, *V* on |ϕ〉, but it depends on $\lambda_{i}$. It can be checked that

(37) $$\sigma =\left(\Phi ⊗1\;l\right)(|\phi \rangle \langle \phi |)=\left(1\;l⊗\mathrm{diag}\left(\sqrt{\lambda }\right)\right)D_{\Phi }\left(1\;l⊗\mathrm{diag}\left(\sqrt{\lambda }\right)\right)$$

and

(38) γ=(R⊗1l)(|ϕ〉〈ϕ|)=1l/d⊗diag(λ).

Next consider $D\left(\Phi ∥R\right)=\sup_{|\phi \rangle \in L\left(X_{A}⊗X_{R}\right)}\mathrm{Tr}\sigma \log\sigma -\mathrm{Tr}\sigma \log\gamma$. Moreover,

(39) $$\begin{array}{cc} \mathrm{Tr}\sigma \log\gamma & =\mathrm{Tr}D_{\Phi }\cdot 1\;l⊗\mathrm{diag}\left(\lambda \log\frac{\lambda }{d}\right)\\ \; & =\mathrm{Tr}{\mathrm{Tr}}_{A}D_{\Phi }\cdot \mathrm{diag}\left(\lambda \log\frac{\lambda }{d}\right)=-H\left(\lambda \right)-\log d \end{array}$$

The above expression reaches minimum for uniform distributed λ and then is equal to Trσlogγ=−2logd. Since σ has spectrum given by μ⊠ν, then

(40) Trσlogσ≃−H(μ⊠ν),

where ⊠ denotes the multiplicative free convolution of measures μ and ν [27]. Assuming maximal entropy H(λ)=log(d) implies ν=δ(1/d), which behaves like an identity in the operation ⊠. Here, δ(1/d) denotes the Dirac distribution (i.e., a distribution with all of its mass localized in 1/d). Hence, we have

(41) H(μ⊠ν)=H(μ),

which gives us

(42) $$D\left(\Phi ∥R\right)=\frac{1}{2}.$$

Now, going back to the entropy of the channel Φ we have

(43) $$H\left(\Phi \right)≃\log\left(d\right)-\frac{1}{2}+o\left(1\right).$$

**Figure 1:**
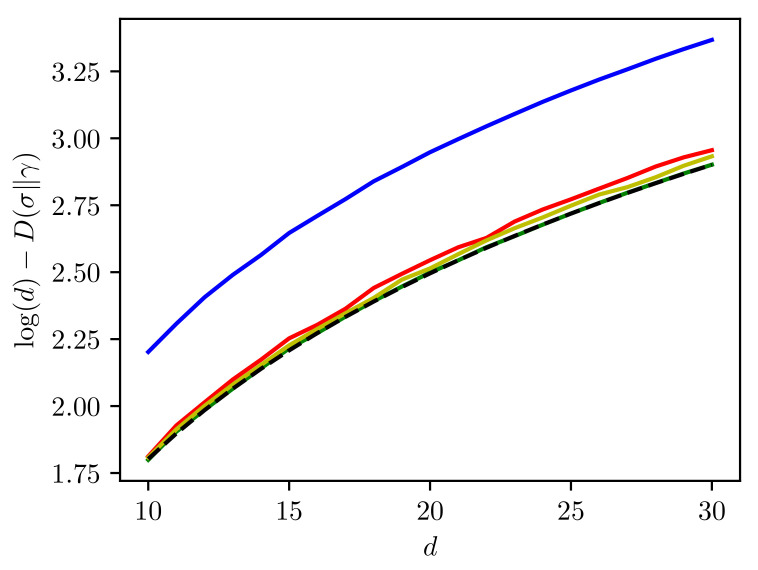
The quantity D(σ∥γ) where σ and γ are as in Equations (37) and (38) respectively. The plots are for ν=Dir(d,1) (red), ν=Dir(2,1) (blue), ν=Dir(d,2) (yellow) and ν=δ(1/d) (green). The dashed line is the quantity $\log\left(d\right)-\frac{1}{2}$.

Let us denote by Dir(d,a) a Dirichlet distribution on a d−1 dimensional simplex with all parameters equal to *a*. Then, the intuition behind our assumption that μ=δ(1/d) is presented in Figure 1. In it, we present the quantity D(σ∥γ) where σ and γ are as in Equations (37) and (38) respectively. The plots are presented for various distributions ν of the Schmidt numbers of the input state |ψ〉. The red line shows the case ν=Dir(d,1), the blue line shows the case when ν=Dir(2,1), the yellow line is the case ν=Dir(d,2) and finally, the green line shows the case ν=δ(1/d). The dashed line is the quantity $\log\left(d\right)-\frac{1}{2}$. As can be seen, the more non-zero Schmidt numbers and the more they are concentrated in the center of the simplex $\Delta_{d-1}$, the closer we get to the quantity we conjecture. Finally, when we choose a deterministic distribution in the center of the simplex, we achieve the optimal value.

## 5. Conclusions

In this paper, we discuss two approaches to entropic quantification of quantum channels. We begin our studies with a formulation of a lemma, which describes a relation between the entropy of quantum channels proposed by Gour and Wilde [18] and entropy of Jamiołkowski matrix of quantum channels [8,9]. We show that both definitions give the same value up to an additive constant in the case of the quantum unital qubit channels. This part of our considerations uses the mathematical language of distinguishability of quantum states and channels. Therefore we assume that obtained results can be used to study resource theories and hypothesis testing. Yet, we need to stress that there exist non-unital channels which saturate the inequality Equation (8), as shown in the discussion after Theorem 1.

We also provide a conjecture backed by numerical experiments that both formulas provide the same results up to an additive constant in the case of large system size.

## References

[1] Devetak I., Winter A. (2005). Distillation of secret key and entanglement from quantum states. Proc. R. Soc. Lond. A.

[2] Berta M., Christandl M., Colbeck R., Renes J.M., Renner R. (2010). The uncertainty principle in the presence of quantum memory. Nat. Phys..

[3] Chitambar E., Gour G. (2019). Quantum resource theories. Rev. Mod. Phys..

[4] Gühne O. (2004). Characterizing entanglement via uncertainty relations. Phys. Rev. Lett..

[5] Oppenheim J., Wehner S. (2010). The uncertainty principle determines the nonlocality of quantum mechanics. Science.

[6] Rastegin A.E. (2016). Separability conditions based on local fine-grained uncertainty relations. Quantum Inf. Process..

[7] Enríquez M., Puchała Z., Życzkowski K. (2015). Minimal rényi–ingarden–urbanik entropy of multipartite quantum states. Entropy.

[8] Roga W., Życzkowski K., Fannes M. (2011). Entropic characterization of quantum operations. Int. J. Quantum Inf..

[9] Roga W., Puchała Z., Rudnicki Ł., Życzkowski K. (2013). Entropic trade-off relations for quantum operations. Phys. Rev. A.

[10] Shaari J.S., Mancini S. (2020). Entropic bounds for unitary testers and mutually unbiased unitary bases. Ann. Phys..

[11] Rudnicki Ł., Puchała Z., Życzkowski K. (2014). Strong majorization entropic uncertainty relations. Phys. Rev. A.

[12] Coles P.J., Piani M. (2014). Improved entropic uncertainty relations and information exclusion relations. Phys. Rev. A.

[13] Rastegin A.E., Życzkowski K. (2016). Majorization entropic uncertainty relations for quantum operations. J. Phys. A Math. Theor..

[14] Kurzyk D., Pawela Ł., Puchała Z. (2018). Conditional entropic uncertainty relations for tsallis entropies. Quantum Inf. Process..

[15] Puchała Z., Rudnicki Ł., Krawiec A., Życzkowski K. (2018). Majorization uncertainty relations for mixed quantum states. J. Phys. A Math. Theor..

[16] Vedral V. (2002). The role of relative entropy in quantum information theory. Rev. Mod. Phys..

[17] Yuan X. (2019). Hypothesis testing and entropies of quantum channels. Phys. Rev. A.

[18] Gour G., Wilde M.M. (2021). Entropy of a quantum channel. Phys. Rev. Res..

[19] Liu Z.-W., Winter A. (2019). Resource theories of quantum channels and the universal role of resource erasure. arXiv.

[20] Katariya V., Wilde M.M. (2021). Geometric distinguishability measures limit quantum channel estimation and discrimination. Quantum Inf. Process..

[21] Fang K., Fawzi O., Renner R., Sutter D. (2020). Chain rule for the quantum relative entropy. Phys. Rev. Lett..

[22] Leditzky F., Kaur E., Datta N., Wilde M.M. (2018). Approaches for approximate additivity of the holevo information of quantum channels. Phys. Rev. A.

[23] Fang K., Fawzi H. (2021). Geometric rényi divergence and its applications in quantum channel capacities. Commun. Math. Phys..

[24] Umegaki H. (1962). Conditional Expectation in an Operator Algebra, IV (Entropy and Information). Kodai Mathematical Seminar Reports.

[25] Haber H.E. Notes on the Matrix Exponential and Logarithm. http://scipp.ucsc.edu/~haber/webpage/MatrixExpLog.pdf.

[26] Nechita I., Puchała Z., Pawela Ł., Życzkowski K. (2018). Almost all quantum channels are equidistant. J. Math. Phys..

[27] Voiculescu D. (1987). Multiplication of certain non-commuting random variables. J. Oper. Theory.

[28] Życzkowski K., Penson K.A., Nechita I., Collins B. (2011). Generating random density matrices. J. Math. Phys..

[29] Puchała Z., Pawela Ł., Życzkowski K. (2016). Distinguishability of generic quantum states. Phys. Rev. A.

[30] Kukulski R., Nechita I., Pawela Ł., Puchała Z., Życzkowski K. (2021). Generating random quantum channels. J. Math. Phys..

